# Screening for Decreased Glomerular Filtration Rate and Associated Risk Factors in a Cohort of HIV-Infected Patients in a Middle-Income Country

**DOI:** 10.1371/journal.pone.0093748

**Published:** 2014-04-03

**Authors:** Patrícia Santiago, Beatriz Grinsztejn, Ruth Khalili Friedman, Cynthia B. Cunha, Lara Esteves Coelho, Paula Mendes Luz, Albanita Viana de Oliveira, Ronaldo Ismério Moreira, Sandra W. Cardoso, Valdilea G. Veloso, José H. Rocco Suassuna

**Affiliations:** 1 STD/AIDS Clinical Research Laboratory, Instituto de Pesquisa Clínica Evandro Chagas-Fundação Oswaldo Cruz, Rio de Janeiro, Brazil; 2 Clinical and Academic Unit of Nephrology, Hospital Universitário Pedro Ernesto, Faculdade de Ciências Médicas, Universidade do Estado do Rio de Janeiro, Rio de Janeiro, Brazil; 3 Department of Pathology, Faculdade de Ciências Médicas, Universidade do Estado do Rio de Janeiro, Rio de Janeiro, Brazil; University of Sao Paulo Medical School, Brazil

## Abstract

With the introduction of combined active antiretroviral therapy and the improved survival of HIV-infected patients, degenerative diseases and drug toxicity have emerged as long-term concerns. We studied the prevalence of decreased glomerular filtration rate (GFR) and associated risk factors in a cohort of HIV-infected patients from a middle-income country. Our cross-sectional study included all adult patients who attended an urban outpatient clinic in 2008. GFR was estimated using the CKD-EPI equation. The prevalence ratio (PR) of decreased GFR (defined as <60 mL/min/1.73 m^2^) was estimated using generalizing linear models assuming a Poisson distribution. We analyzed data from 1,970 patients, of which 82.9% had been exposed to ART. A total of 249 patients (12.6%) had a GFR between 60 and 89 mL/min/1.73 m^2^, 3.1% had a GFR between 30 and 59, 0.3% had a GFR between 15 and 29, and 0.4% had a GFR <15. Decreased GFR was found in only 74 patients (3.8%). In the multivariate regression model, the factors that were independently associated with a GFR below 60 mL/min/1.73 m^2^ were as follows: age ≥50 years (PR = 3.4; 95% CI: 1.7–6.8), diabetes (PR = 2.0; 95% CI: 1.2–3.4), hypertension (PR = 2.0; 95% CI: 1.3–3.2), current CD4+ cell count <350 cells/mm3 (PR = 2.1; 95% CI: 1.3–3.3), past exposure to tenofovir (PR = 4.7; 95% CI: 2.3–9.4) and past exposure to indinavir (PR = 1.7; 95% CI: 1.0–2.8). As in high-income countries, CKD was the predominant form of kidney involvement among HIV-infected individuals in our setting. The risk factors associated with decreased glomerular filtration were broad and included virus-related factors as well as degenerative and nephrotoxic factors. Despite the potential for nephrotoxicity associated with some antiretroviral drugs, in the short-term, advanced chronic renal disease remains very rare.

## Introduction

The introduction of combined active antiretroviral therapy (cART) for HIV infection was followed by a significant decline in the number of deaths attributable to opportunistic infections. As the survival of chronically HIV-infected individuals increased, so did the prevalence of major chronic degenerative disorders, including metabolic, cardiovascular, renal, and bone-related conditions. For example, the widespread use of cART caused a marked drop in the incidence of rapidly progressive HIV-associated nephropathy (HIVAN), but HIV infection is now associated with indolently developing chronic kidney disease (CKD) [1], [2]. Currently, renal disease risk in patients with HIV infection appears to be compounded by ethnicity, chronic comorbidities, concurrent viral infections, and the use of antiretroviral drugs with nephrotoxic potential [2], [3]. Regardless of the underlying cause, CKD is considered a significant independent risk factor for mortality among HIV-infected patients in both developed and resource-limited settings [4]–[7]. Given these adverse associations, all HIV-infected persons are advised to undergo kidney function evaluation [8].

In 1991, the Brazilian Ministry of Health launched a pioneer program to provide free access to antiretroviral treatment for all HIV-infected individuals as needed. A marked drop in morbidity and mortality rewarded Brazil’s unique position of a middle-income economy with more than 200.000 patients receiving cART [9]. While successful, increasing patient life expectancy and HIV infection prevalence is anticipated to place a growing burden on limited local specialized human resources and health care logistics. Because the first step toward preparation and planning involves collecting evidence, the objectives of this study were to screen for decreased glomerular filtration rate (GFR) and associated risk factors in an outpatient cohort of HIV-infected patients followed at Instituto de Pesquisa Clinica Evandro Chagas (IPEC)/Fiocruz, a referral center for HIV care and research in the city of Rio de Janeiro, Brazil.

## Methods

### Ethics Statement

The project was submitted to the Institutional Review Board of Instituto de Pesquisa Clínica Evandro Chagas - Fundação Oswaldo Cruz and approved on Sept 13, 2010 as # 044/2010. A translation of the conclusion of the board is read as follows: “In substitution to written informed consent, following declaration #196 of 1996 from the Brazilian National Health Council, the investigators in charge (PS and BG) subscribed to a term of agreement pledging confidentiality and anonymity of the participants of the study”. The project was not specifically funded.

### Cohort Identification and Study Design

The IPEC clinical HIV service has been providing patient care since 1986. The HIV cohort database was started in 1998. Longitudinal data are updated regularly using clinic and inpatient clinical documentation, laboratory results, and pharmacy records. Medical providers and support staff record clinical information, including prescriptions for antiretroviral drugs. Trained data abstractors collect relevant information onto standardized forms for further processing.

We performed a cross-sectional study including all patients aged 18 years or older who attended the IPEC outpatient clinic and had at least one serum creatinine evaluation during the calendar year of 2008. HIV infection was diagnosed according to the Brazilian Ministry of Health criteria [10]: either two ELISA tests or two rapid tests followed by a confirmatory test, either a Western blot or immunofluorescence assay. Creatinine was measured with the Roche enzymatic assay at the IPEC Safety Laboratory, which is certified by the American College of Pathology. We excluded patients with missing ethnicity data and those whose creatinine results were obtained during or up to six weeks after admission to a hospital. The outcome of interest was the estimated glomerular filtration rate (eGFR), calculated from a single fasting creatinine measurement using the CKD-EPI equation. The GFR results were stratified according to the NKF-K/DOQI staging system. All of the patients classified in stages 3 through 5 (i.e., with a GFR <60 mL/min/1.73 m^2^) were considered to have a decreased GFR.

The data that were obtained concurrently with the assessment of renal function and evaluated as explanatory variables for reduced GFR included gender, age, ethnicity, HIV transmission group, AIDS defining illness (according to the 1993 AIDS CDC definition**)**, time since the HIV infection diagnosis, co-infection with the hepatitis B (HBV) virus (defined as a positive HBsAg test), co-infection with the hepatitis C (HCV) virus (defined as positive anti-HCV serology), diabetes (defined as the use of anti-diabetic drugs or a record in the patient’s chart), hypertension (defined as a record in the chart or the use of antihypertensive therapy), hyperlipidemia (defined by the prescription of lipid-lowering drugs or documentation in the chart), current HIV1-RNA plasma viral load, current CD4+ cell count, nadir CD4+ cell count, past and current ARV exposure, and exposure to selected nephrotoxins in the three years prior to the GFR assessment (aciclovir, valaciclovir, ganciclovir, gentamicin, amikacin, amphotericin B, and meglumine antimoniate).

### Statistical Analysis

The prevalence ratio (PR) of decreased GFR was estimated with generalizing linear models created assuming a Poisson distribution with logarithmic link function and robust variance. The covariates that were significantly associated with renal dysfunction in the univariate analysis (p<0.10) were entered in the multivariate models. Given the low prevalence of the study outcome, the variables were retained if they met the condition p≤0.10 in a backward elimination strategy and if they were considered potential confounders (e.g., when removed, a change equal to or greater than 20% in the prevalence ratio of any other variable of the model was observed). All of the analyses were performed using STATA/SE version 10.1.

## Results

### Study Population

Between January 1 and December 31, 2008, 2,345 patients were seen at the IPEC outpatient clinic. A total of 375 patients (16%) were excluded from further analyses for the following reasons: lack of serum creatinine testing (310), creatinine testing performed exclusively during hospitalization or within 6 weeks after hospital discharge (to prevent the inclusion of patients with acute kidney injury) (63), and lack of ethnicity data (thus interfering with the estimation of GFR by the CKD-EPI equation) (2). We analyzed data from 1,970 patients. The median age was 41.6 (interquartile range [IQR] 34.0–48.2) years; 63.6% were male and 57.1% were white (Table 1). The patients were followed at IPEC for 10,456 person-years.

**Table 1:** Demographic and clinical characteristics of the study patients.

Characteristic		Total Cohortn (%)	eGFR ≥60n (%)	eGFR <60n (%)
**Gender**	Male	1252 (63.6%)	1203 (63.4%)	49 (66.2%)
	Female	718 (36.4%)	693 (36.6%)	25 (33.8%)
**Ethnic origin**	Non white	845 (42.9%)	815 (43.0%)	30 (40.5%)
	White	1125 (57.1%)	1081 (57.0%)	44 (59.5%)
**Age (years)**	<40	879 (44.6%)	867 (45.7%)	12 (16.2%)
	40–50	698 (35.4%)	674 (35.5%)	24 (32.4%)
	>50	393 (19.9%)	355 (18.7%)	38 (51.4%)
**Diabetes mellitus**	Present	182 (9.3%)	162 (8.6%)	20 (27.0%)
**Hypertension**	Present	523 (26.6%)	483 (25.5%)	40 (54.1%)
**Hyperlipidemia**	Present	913 (46.9%)	870 (46.4%)	43 (58.9%)
**Hepatitis B surface antigen**	Detectable	58 (2.9%)	56 (3.0%)	2 (2.7%)
**Hepatitis C antibody**	Detectable	119 (6.0%)	111 (5.9%)	8 (11.0%)
**Time since first HIV-positive serology (IQR)**	Months	78.5 (29.1–136.2)	77.1 (28.9–134.2)	117.7 (50.7–158.7)
**AIDS defining illness**	Present	843 (42.8%)	804 (42.4%)	39 (52.7%)
**Nadir CD4+ count <200 cells/mm^3^**	Present	1008 (51.7%)	961 (51.1%)	47 (67.1%)
**CD4+ count <350 cells/mm^3^ within 6 months of index creatinine**	Present	610 (31.9%)	574 (31.2%)	36 (50.7%)
**HIV viral load within 6 months of index creatinine**	Detectable	558 (29.5%)	535 (29.5%)	22 (31.9%)
**Exposure to tenofovir**	Never	1162 (59.0%)	1133 (59.8%)	29 (39.7%)
	Prior	63 (3.2%)	50 (2.6%)	13 (17.8%)
	Current	744 (37.8%)	713 (37.6%)	31 (42.5%)
**Exposure to atazanavir and/or lopinavir**	Never	1111 (56.4%)	1088 (57.4%)	23 (31.5%)
	Prior	312 (15.8%)	298 (15.7%)	14 (19.2%)
	Current	546 (27.7%)	510 (26.9%)	36 (49.3%)
**Exposure to indinavir**	Prior	305 (15.5%)	278 (14.7%)	27 (37.0%)
**Exposure to non-ARV nephrotoxic drugs** *	Present	338 (17.5%)	324 (17.4%)	14 (19.7%)

*Patients to whom it was prescribed one of these in the last three years: guanosine analogue antiviral drugs (aciclovir, ganciclovir, valaciclovir), aminoglycosides (amikacin, gentamicin), amphotericin B preparations, and meglumine antimoniate.

Hyperlipidemia, hypertension, and diabetes mellitus were present in 46.9%, 26.6%, and 9.3% of the patients, respectively. The median time since the diagnosis of HIV infection was 78.5 (IQR 29.1–136.2) months, and 42.8% met the clinical definition for AIDS. The median nadir and current CD4+ cell counts were 189 (IQR 78–308) and 460 (IQR 307–650) cells/mm^3^, respectively. The plasma viral load was undetectable in 70.5% of the subjects.

Overall, 1,634 patients (82.9%) had been exposed to ART, with a mean cumulative exposure (MCE) of 67.7 months (SE: 1.3) and a total of 9206 person-years of follow up. Of the patients, 807 (41.0%) had been exposed to tenofovir (MCE 25.0±19.3 months), 305 (15.5%) had been exposed to indinavir (MCE 31.8±26.5 months), 490 (24.9%) had been exposed to atazanavir (MCE: 25.7±18.8 months), and 516 (26.2%) had been exposed to lopinavir (MCE: 27.4±21.3 months).

At the time of evaluation, 744 patients were on tenofovir (TDF), and 67.5% (n = 502) had been exposed for at least one year. Among the patients exposed to TDF in the past (n = 63), 52.4% had been exposed for more than one year. A total of 860 patients were exposed to atazanavir and/or lopinavir (ATV⋁LPV); 546 were currently using one of these drugs, and of these patients, 77.7% (n = 424) had at least one year of exposure. Among the patients exposed to ATV⋁LPV in the past (n = 314), 72.9% had been exposed for at least one year. Prior exposure to potentially nephrotoxic non-ARV drugs was recorded in 345 subjects (17.5%) as follows: aciclovir, 13.2%; valaciclovir, 4.9%; ganciclovir, 0.6%; amikacin, 0.2%; gentamicin, 0.1%; amphotericin B preparations, 0.1%; and meglumine antimoniate, 0.1%.

The median eGFR for the entire cohort was 111.4 mL/min/1.73 m^2^. Table 2 presents the categories of eGFR prevalence according to the NKF-K/DOQI stages. Of the 1,970 patients, 1,647 (83.6%) had an eGFR above 90 mL/min/1.73 m^2^, 249 patients (12.6%; 95% CI: 11.2–14.2) had an eGFR between 60 and 89 mL/min/1.73 m^2^, 61 patients (3.1%; 95% CI: 2.4–4.0) had an eGFR between 30 and 59 mL/min/1.73 m^2^, six patients (0.3%; 95% CI: 0.1–0.7) had an eGFR between 15 and 29 mL/min/1.73 m^2^, and seven patients (0.4%; 95% CI: 0.1–0.7) had an eGFR below 15 mL/min/1.73 m^2^. Notably, 74 patients (3.8%; 95% CI: 3.0–4.7) fulfilled the primary outcome criterion of an eGFR below 60 mL/min per 1.73 m^2^.

**Table 2:** Prevalence of estimated GFR (eGFR) categories in the IPEC/FIOCRUZ cohort of HIV-infected patients.

Glomerular filtration rate (mL/min per 1.73 m^2^)*	n	% (95% CI)
≥90	1647	83.6 (81.9–85.2)
60–89	249	12.6 (11.2–14.2)
30–59	61	3.1 (2.4–4.0)
15–29	6	0.3 (0.1–0.7)
<15	7	0.4 (0.1–0.7)

*Estimated by the CKD-EPI equation.

Table 1 also shows that in comparison with the patients with GFR ≥60 mL/min per 1.73 m^2^, the patients with decreased GFR were more frequently aged 50 years or older (51.4% vs. 18.7%) and had higher prevalence rates of hyperlipidemia (58.9% vs. 46.6%), hypertension (54.1% vs. 25.5%), diabetes (27.0% vs. 8.6%), and positive anti-HCV serology (11.0% vs. 5.9%). These patients also had been diagnosed with HIV for a longer time, with a median time of 117.7 (IQR 50.7–158.7) vs. 77.1 (IQR 28.9–134.2) months, and they were more likely to have an AIDS diagnosis (52.7% vs. 42.4%) and a current CD4+ count below 350 cells/mm^3^ (50.7% vs. 31.2%).

Exposure to TDF (60.3% vs. 40.2%), current (42.5% vs. 37.6%) and previous use of TDF (17.8% vs. 2.6%), and exposure for at least one year (43.0% vs. 26.5%) were more common among the patients with decreased GFR. The same trend was observed for exposure to ATV⋁LPV (68.5% vs. 42.6%), current use of ATV⋁LPV (49.3% vs. 26.9%), and exposure to these drugs for at least one year (58.9% vs. 32.2%). Past exposure to indinavir was also more common among the patients with reduced GFR (37% vs. 14.7%). Exposure to potentially nephrotoxic non-ARV drugs was observed in 19.7% of the patients with GFR <60 mL/min/1.73 m^2^ vs. 17.4% of the patients with GFR ≥60 mL/min/1.73 m^2^ (Table 1).

In the univariate analysis, age, diabetes, hypertension, hyperlipidemia, hepatitis C, time since the HIV diagnosis, opportunistic diseases, current CD4+ count, indinavir exposure, and past and current exposure to TDF and ATV⋁LPV were significantly and positively associated with the primary outcome (Table 3).

**Table 3:** Factors associated with decreased GFR (<60 mL/min per 1.73 m^2^) in HIV-infected patients of the IPEC/FIOCRUZ Cohort.

Characteristic		Crude PR(95% CI)	*p*-value	Adjusted PR(95% CI)	*p*-value
**Gender**	Male	1.00			
	Female	0.89 (0.55–1.43)	0.628	–	–
**Ethnic origin**	Non white	1.00			
	White	0.91 (0.58–1.43)	0.677	–	–
**Age (years)**	<40	1.00		1.00	
	40–50	2.52 (1.27–5.00)	0.008	1.5 (0.7–2.9)	0.287
	>50	7.08 (3.74–13.41)	<0.001	3.4 (1.7–6.8)	0.001
**Diabetes mellitus**	No	1.00		1.00	
	Yes	3.63 (2.23–5.93)	<0.001	2.0 (1.2–3.4)	0.009
**Hypertension**	No	1.00		1.00	
	Yes	3.25 (2.08–5.08)	<0.001	2.0 (1.3–3.2)	0.004
**Hyperlipidemia**	No	1.00			
	Yes	1.62 (1.03–2.57)	0.038	–	–
**Hepatitis B surface antigen**	No	1.00			
	Yes	0.92 (0.23–3.64)	0.901	–	–
**Hepatitis C antibody**	No	1.00			
	Yes	1.91 (0.94– 3.89)	0.074	–	–
**Months since first HIV-positive serology (IQR)**		1.06 (1.02–1.1)	0.002	–	–
**AIDS defining illness**	No	1.00			
	Yes	1.49 (0.95–2.33)	0.081	–	–
**Nadir CD4+ count (cells/mm^3^)**	≥200	1.00			
	<200	1.91 (1.17–3.12)	0.01	–	–
**CD4+ count within 6 months of index creatinine (cells/mm^3^)**	≥350	1.00		1.00	
	<350	2.19 (1.39–3.46)	0.001	2.1 (1.3–3.3)	0.003
**HIV viral load within 6 months of index creatinine**	Detectable	1.00			
	Undetectable	0.89 (0.54–1.47)	0.658	–	–
**Exposure to tenofovir**	Never	1.00		1.00	
	Prior	8.27 (4.52–15.11)	<0.001	4.7 (2.3–9.4)	<0.001
	Current	1.67 (1.01–2.75)	0.044	1.1 (0.6–2.0)	0.713
**Exposure to atazanavir and/or lopinavir**	Never	1.00		1.00	
	Prior	2.17 (1.13–4.16)	0.020	0.9 (0.4–2.0)	0.746
	Current	3.18 (1.91–5.32)	<0.001	1.7 (0.9–3.1)	0.090
**Prior exposure to indinavir**	No	1.00		1.00	
	Yes	3.20 (2.02–5.07)	<0.001	1.7 (1.0–2.8)	0.054
**Exposure to non-ARV nephrotoxic drugs**	No	1.00			
	Yes	1.16 (0.65–2.06)	0.614	–	–

In the multivariate adjusted Poisson regression model, the following factors were significantly associated (p<0.05) with a GFR <60 mL/min per 1.73 m^2^: age ≥50 years (PR = 3.4; 95% CI: 1.7–6.8), diabetes (PR = 2.0; 95% CI: 1.2–3.4), hypertension (PR = 2.0; 95% CI: 1.3–3.2), current CD4+ cell count <350 cells/mm^3^ (PR = 2.1; 95% CI: 1.3–3.3), and past exposure to TDF (PR = 4.7; 95% CI: 2.3–9.4) and IDV (PR = 1.7; 95% CI: 1.0–2.8). Among the patients using ATV or LPV at the time of creatinine evaluation, the prevalence of decreased GFR was higher than the prevalence of those never exposed to such drugs, although this association was not statistically significant (PR = 1.7; 95% CI: 0.9–3.1; p = 0.09) (Table 3).

## Discussion

The main findings of our study were as follows: (1) as far as we know, our study investigated the prevalence of decreased GFR in the largest cohort of HIV patients highly exposed to antiretroviral drugs from a middle-income country; (2) we found that only 3.8% of the participants had a GFR below 60 mL/min per 1.73 m^2^ as estimated by the CKD-EPI equation; and (3) the results indicate that a decreased GFR was positively and independently associated with markers of the severity of HIV infection as well as with other chronic degenerative diseases and exposure to nephrotoxic antiretroviral drugs.

Large cohort studies (i.e., with more than one thousand members) investigating the prevalence of CKD in HIV-infected individuals have been conducted with either cART-treated patients from high-income countries [11]–[22] or predominantly treatment-naïve subjects from low-income settings [23]–[25]. Healthcare in middle-income countries fits between these two extremes. While low-income countries still struggle to meet the needs of their people, middle-income economies have already developed the ability to deliver basic health services but face hurdles regarding coverage, priority setting, financing, and efficiency. With regard to HIV treatment, despite undeniable progress, middle-income countries exhibit marked differences in access to cART and treatment consistency [26]. The epidemiological features of our cohort, including the prevalence of CKD risk factors, are akin to those in higher-income countries (e.g., the predominance of urban white males in early middle age (median age 41.6 years), access to cART, and a moderate prevalence of chronic comorbidities).

Decreased GFR was uncommon (3.8%) among the HIV-infected patients in our cohort. Differences in the baseline sociodemographic data, the cohort inclusion criteria and the accrual period as well as exposure to different antiretroviral medications preclude exact comparisons among published reports. Nevertheless, data from the high-income country cohorts mentioned above show that the median ages and prevalence rates of CKD (defined by GFR <60 mL/min/1.73 m^2^) for the three main creatinine-based GFR estimation methods were as follows: an age of 42.8 years and a CKD prevalence of 4.2% for CG [17], [19], an age of 39.3 years and a CKD prevalence of 4.5% for MDRD [11]–[16], [20], [27], and an age of 41.3 years and a CKD prevalence of 4.5% for CKD-EPI GFR [18], [19], [21], [22].

Notably, 16.4% of the members of the cohort had estimated GFRs below 90 mL/min/1.73 m^2^. Previously, similar double figure rates have been highlighted as an indication of a high burden of CKD in HIV-infected cohorts [19], [28], [29]. However, a sizable proportion of healthy normal adults have eGFR values within the range of 60 to 89 mL/min/1.73 m^2^. Consequently, it is feared that such a high cut-off inappropriately inflates the prevalence of CKD [30], [31]. In any case, even this threshold has been shown as an independent risk factor for cardiovascular mortality in apparently healthy adults [32].

Concerns about the over-diagnosis of CKD are extensive for our chosen eGFR cut-off of 60 mL/min/1.73 m^2^ [33], [34]. Nevertheless, this boundary is widely used for screening purposes [35], [36]. More importantly, a single eGFR value under 60 mL/min/1.73 m^2^ is independently associated with an increased risk of cardiovascular [32], [37]–[40] and all-cause mortality [37], [39]. Furthermore, most cases of false-positive CKD labeling involve older individuals, mostly females with low muscle mass. We studied a targeted group of young to middle-aged men with known risk factors for chronic kidney damage, therefore enhancing the positive predictive value and minimizing the negative predictive value of the eGFR test [30], [31].

Cross-sectional studies in healthy individuals show that directly measured glomerular filtration rated are lower in older adults than younger adults [41] and that this gap is magnified when GFR is estimated with creatinine-based equations [42]. We, like others [11], [15], [16], [19], [21], [27], did find that a lower eGFR was independently associated with increasing age. This finding was particularly relevant given the rising incidence of HIV infection in older adults [43], [44] and the remarkable success of cART, which is allowing younger infected adults to age [45]. In addition, HIV-associated inflammation, exposure to potentially toxic antiretroviral drugs, accelerated aging, and chronic comorbidities contribute to the premature occurrence of “non-AIDS” degenerative diseases, including CKD [46], [47]. With regard to this matter and in agreement with previous reports [3], [15], [17], [22], [48], we noted that both diabetes and hypertension were significantly and independently associated with a low eGFR.

Previous studies have provided evidence that cART controls kidney cell damage and preserves renal function [49] and that its withdrawal is associated with HIVAN and kidney function deterioration [50]. In our cohort, 18.1% of the patients receiving cART had detectable viral loads, and 32% had a current CD4+ cell count below 350, indicating some degree of uncontrolled infection, due to either viral resistance or non-adherence. Overall, our patients had fairly advanced HIV disease, as shown by the median nadir of 189 CD4+ cells/mm^3^ and by the fact that 42.8% had a previous AIDS defining illness. Against this background, we did not find an association between reduced GFR and either of these variables, but we noticed that a reduced GFR was significantly associated with a current CD4+ count below 350 cells/mm^3^, a marker of incomplete immune reconstitution.

The rationale behind cART is to administer multiple drugs targeting different stages of HIV cell entry and the cell life cycle to achieve long-lasting viral suppression. This strategy exposes patients to multiple potential drug interactions and entails a lifelong risk of drug toxicity, including nephrotoxicity [51]. We observed that the past use of tenofovir and indinavir was associated with a decreased glomerular filtration rate. An association between current atazanavir and/or lopinavir use and decreased glomerular filtration rate was also observed but it did not reach statistical significance (p = 0.09).

Tenofovir was used by 41% of our patients, and 84.8% of those patients were still using the drug. Even after accounting for demographics, HIV-related factors, comorbidities, and other antiretroviral drugs, past TDF use remained independently associated with low GFR compared with those with no previous exposure. This result suggests that kidney damage and dysfunction do not quickly reverse after the exposure ends. In fact, among the patients who discontinue TDF because of renal impairment, only a minority returns to their pre-TDF GFR [52]. Furthermore, data from a large cohort in the US showed that past users of TDF remained at an increased risk of proteinuria and rapid renal function decline compared with those never exposed to TDF [2]. Because TDF is a first-line treatment for HIV infection that is increasingly used in all antiretroviral regimens and in pre-exposure prophylaxis, attention to the early signs of renal toxicity, perhaps even above the 60 mL/min/1.73 m^2^ cutoff, is necessary [52].

Contrary to previous use, the current use of TDF was not associated with a decreased GFR. This finding differs from other reports that did find such an association [19], [20]. Several possible underlying explanations can be given for this result, including differences in study definitions, in the background characteristics of the study populations, and in the length of exposure to tenofovir, among others. Nevertheless, we believe that the broadly reported association between TDF and renal dysfunction [2], [53] led to careful and repeated measurements of serum creatinine, resulting in earlier withdrawal and prevention of unchecked toxic exposure to TDF. In fact, recent data from the D:A:D study showed that decreased GFR was associated with a significant rate of TDF discontinuation, which prevented further deterioration of renal function. Interestingly, this behavior was not replicated when the GFR declined in patients exposed to other antiretroviral agents [54].

Both indinavir and atazanavir have been associated with crystalluria, crystal nephropathy, nephrolithiasis, and CKD in previous studies [17], [55]. In our cohort, past users of indinavir had a higher risk of GFR decline that reached borderline significance (PR = 1.7; 95% CI: 1.0–2.8). In recent years, the newer protease inhibitors, particularly atazanavir and lopinavir, have replaced indinavir. Contrary to the above mentioned studies, past or current use of atazanavir or lopinavir was not significantly associated with a GFR below 60 mL/min/1.73 m^2^. However, in the multivariate regression model, there was a trend toward an association of current use of ATV⋁LPV and low GFR (PR = 1.7; 95% CI: 0.9–3.1). It is possible that the low prevalence of the decreased GFR end-point in the cohort contributed to this negative finding. Further investigation is necessary to delineate the impact of these drugs on kidney function.

This study does have several limitations. First, as in all cohorts, the sample includes adherent individuals with extensive and complicated medical histories that may obscure the associations of interest [56]. Second, because of the cross-sectional design, we can draw associations between events but cannot establish temporal sequences. Third, the diagnosis of low GFR was based on an isolated creatinine measurement. In spite of this, as discussed above, this strategy has been used widely in screening investigations related to CKD and associated risk factors [35], [36], [57], [58], and a single eGFR below 60 mL/min/1.73 m^2^ is associated with cardiovascular [32], [37]–[40] and non-vascular mortality [37], [39]. We were also particularly careful to exclude patients who developed acute kidney injury by excluding any creatinine measurement obtained during a period of hospitalization or up to six weeks thereafter. Fourth, given the low prevalence of the low GFR outcome in our cohort, we may have missed factors associated with renal disease. Finally, kidney disease in subjects with eGFR values above 60 mL/min/1.73 m^2^ may have been underestimated because we lacked data on proteinuria and renal tubular involvement, both early and significant indicators of renal disease in HIV patients.

This study also had multiple strengths. It was conducted at a referral center for HIV/AIDS care and research in one of the epicenters of the Brazilian AIDS epidemic on a very well characterized cohort with mixed ethnic background and universal access to first-line NNRTI-based regimens, as well as free access to PI-based regimens for subsequent treatment steps after first- and second-line virological failure. Additionally, we employed the recently developed CKD-EPI equation, which provides a more accurate estimate of GFR than other creatinine-based equations [59], an observation that was also confirmed in patients with HIV infection [60].

In summary, in this well-characterized cohort of Brazilian patients with HIV/AIDS with long-term exposure to cART, an age over 50 years, a current CD4 count less than 350 cells/mm^3^, diabetes, hypertension, previous use of tenofovir, and previous use of indinavir were associated with a decreased glomerular filtration rate (p<0.05). The current use of atazanavir or lopinavir showed a weaker association with the outcome (p = 0.09), and this result may be related to the low prevalence of the outcome and the study population size. We were able to confirm that the current risk factors for CKD in HIV-infected individuals are broad and include virus-related factors as well as degenerative and nephrotoxic factors. With long-term exposure to cART, within the context of premature aging and cumulative chronic comorbidities, cumulative exposure to even low-grade nephrotoxins may eventually prove to have deleterious effects. Nevertheless, it is reassuring that despite the potential for nephrotoxicity from some pivotal antiretroviral drugs, in the short-term, advanced renal disease remains very rare.
